# A Single-Center Retrospective Study on the Bacteriological Profile of Breast Abscesses

**DOI:** 10.7759/cureus.82173

**Published:** 2025-04-13

**Authors:** Aiswerya Shankar, Magesh Chandran, Madan Sundar

**Affiliations:** 1 General Surgery, Sree Balaji Medical College Hospital, Chennai, IND; 2 Surgery, Bharath Institute of Higher Education and Research, Chennai, IND

**Keywords:** lactational breast abscess, lactational mastitis, methicillin resistant staphylococcus aureus (mrsa), nonpuerperal breast abscess, staph-aureus

## Abstract

Introduction: Breast abscesses are a common clinical condition, primarily affecting lactating women as a result of mastitis. It can also occur in non-lactating women due to a variety of factors such as diabetes, immunosuppression, and trauma. The microbiological etiology of breast abscesses is important for determining effective antibiotic therapy and preventing complications. This study aims to identify the microbiological profile of breast abscesses, characterize the common pathogens, and analyze their antibiotic resistance patterns.

Methods: A retrospective analysis was conducted on 108 patients diagnosed with breast abscesses between June 2023 and January 2025. Hospital records were retrieved and reviewed. Demographic data, microbiological findings and antibiotic treatment outcomes were extracted. The primary objective was to identify the main pathogens causing breast abscess. The secondary objective was to understand the antibiotic resistance patterns and the prevalence of coagulase-negative staphylococci (CoNS) in breast abscesses. Microbiological cultures were obtained from abscess aspirates or purulent drainage material. The bacterial isolates were identified using standard microbiological techniques. Antibiotic susceptibility testing was performed using the disk diffusion method.

Results: The mean age of participants in the study was 26±2 years. Of the 108 women, approximately 84% had lactational breast abscesses, and the remaining 16% had non-lactational breast abscesses. The most frequently isolated microorganism was *Staphylococcus aureus* (41.67%), with 15% of isolates being methicillin-resistant (MRSA). Other common pathogens included *Streptococcus pyogenes* (13.89%), *Escherichia coli* (9.26%), and *Enterococcus faecalis* (7.41%). Polymicrobial infections, including both aerobic and anaerobic organisms, were identified in 8.33% of cases. Antibiotic resistance was notably high for *Staphylococcus aureus*, *Escherichia coli* (10% extended-spectrum beta-lactamase (ESBL)-producing), and *Enterococcus faecalis* (5% vancomycin-resistant). Five patients (4.63%) had no microbial growth. The majority of patients were treated with empirical antibiotics, and therapy was adjusted based on culture results, with good clinical outcomes in most cases.

Conclusion: The microbiological profile of breast abscesses is diverse, with *Staphylococcus aureus* being the predominant pathogen, followed by *Streptococcus pyogenes* and *Escherichia coli*. Antibiotic resistance, particularly in MRSA, poses a significant challenge in treatment. Empiric antibiotic therapy should be tailored according to local resistance patterns to ensure effective treatment and reduce the risk of complications.

## Introduction

Breast abscess is a localized collection of pus within the breast tissue, usually caused by bacterial infections [1]. Although this condition predominantly affects lactating women, non-lactating women also develop breast abscesses. It is often initiated by an infection of the mammary gland. Granulomatous mastitis may develop in the setting of poor drainage of milk, blocked milk ducts, trauma to the breast and may progress if left untreated [1]. Infections of the mammary gland are most common after postpartum, making it more common in lactating women [2]. However, breast abscesses can also occur in non-lactating individuals, typically due to conditions such as diabetic mastitis, immunosuppression, local infection due to skin flora or the presence of foreign bodies like breast implants [1]. Poor hygiene, smoking, and underlying chronic illnesses like diabetes are significant risk factors for inflammation of the periductal region and abscess in non-lactating women [2].

The clinical presentation of a breast abscess typically includes pain, swelling, erythema, and tenderness in the affected area, often accompanied by fever and a localized increase in temperature [3]. The management of breast abscesses generally involves drainage, either through needle aspiration or surgical incision guided by ultrasound, and the administration of appropriate antimicrobial therapy based on the isolated pathogens [4, 5]. Microbiological identification is crucial to guide antibiotic therapy and improve patient outcomes. The most common pathogens responsible for breast abscesses are *Staphylococcus aureus *(*S. aureus*), particularly methicillin-resistant (MRSA), followed by *Enterococcus* species and coagulase-negative staphylococci (CoNS) [1]. However, there is a significant regional variation in the microbial etiology of breast abscesses, emerging pathogens and their antibiotic resistance patterns. This retrospective study aims to provide a comprehensive understanding of the microbiological profile of breast abscesses in a specific cohort and to understand their antibiotic resistance patterns.

## Materials and methods

Study design

This retrospective study was conducted at a tertiary care hospital over a period of one year and 6 months from June 2023 to January 2025 in the Department of General Surgery of Sree Balaji Medical College and Hospital, Chennai, India. Data were obtained from hospital medical records within the specified time frame. The sample size consisted of 108 patients (using convenience sampling) diagnosed with breast abscess. Informed consent was obtained from the participants.

Methods

To include a diverse group of women with breast abscesses, both lactating and non-lactating women who had been diagnosed with breast abscesses through diagnostic imaging, such as ultrasonography or mammography, were a part in the study. Additionally, patients needed to have microbiological confirmation of bacterial or fungal organisms from the pus samples collected. Furthermore, it was essential for patients to have complete clinical and microbiological records available for review, ensuring that the data collected for analysis was thorough and accurate. The study excluded patients who were younger than 18 years old. Patients with incomplete data records (incomplete history/presenting features) were excluded to maintain the integrity of the study’s findings. 

Of the 108 women who were identified as potential candidates for inclusion, the study population was divided into two groups based on the type of breast abscess. Ninety women were classified as having lactational breast abscesses, which were grouped under Group 1, while eighteen women had non-lactational breast abscesses, placing them in Group 2. This division allowed for a comparative analysis between the two groups, focusing on the differences in clinical presentation, microbiological findings, and treatment outcomes. By examining both lactating and non-lactating women, the study aimed to provide comprehensive insights into the factors influencing the development and treatment of breast abscesses across different patient populations.

Data collection

Data for this study were collected retrospectively from a combination of hospital medical records and microbiology laboratory records. The data collection process focused on a variety of key factors, including patient demographic details, clinical presentation, and the treatment administered. Based on the presentation, symptoms and signs, a diagnosis of breast abscess was made. The course of treatment, either ultrasound-guided needle drainage or open incision and drainage, was based on the surgeon. Additionally, the pus culture aspirates were sent for microbiological identification and antibiotic susceptibility testing. All of this information was systematically collected and thoroughly analyzed to draw meaningful conclusions regarding the clinical and microbiological aspects of the cases. To process the microbiological samples, standard bacteriological methods were employed. These methods included Gram staining, for identifying the bacterial species and cultures, which allowed for the isolation of the organisms present in the samples. Aerobic bacteria in breast abscesses were cultured on blood or MacConkey agar and incubated at 37°C in oxygen-rich conditions for 18-24 hours. Anaerobic bacteria require specialized media like anaerobic blood agar, incubated in sealed jars or chambers under oxygen-free conditions for 48-72 hours. Antibiotic susceptibility testing was an essential part of the study and was carried out using the Kirby-Bauer disk diffusion method. This method involves placing antibiotic-impregnated paper disks on a bacterial culture plate and measuring the zone of inhibition, indicating the effectiveness of specific antibiotics against the isolated bacteria. Analyzing these susceptibility patterns helps assess the resistance profile of the organisms and determine the most effective treatment options. Contamination was minimized by using sterile equipment and proper sample collection. Strict aseptic techniques and usage of selective media help prevent external contamination during culture.

Statistical analysis

The data collected in this study were analyzed using descriptive statistics to provide a clear overview of the results. To assess the central tendency and variability of the data, statistical analysis was performed using the mean and standard deviation. The frequency of bacterial pathogens isolated from the breast abscess samples was calculated as a percentage of the total number of cases. Additionally, the antibiotic resistance profiles of the isolated bacterial strains were carefully analyzed to determine the prevalence of resistant strains.

## Results

**Figure 1:**
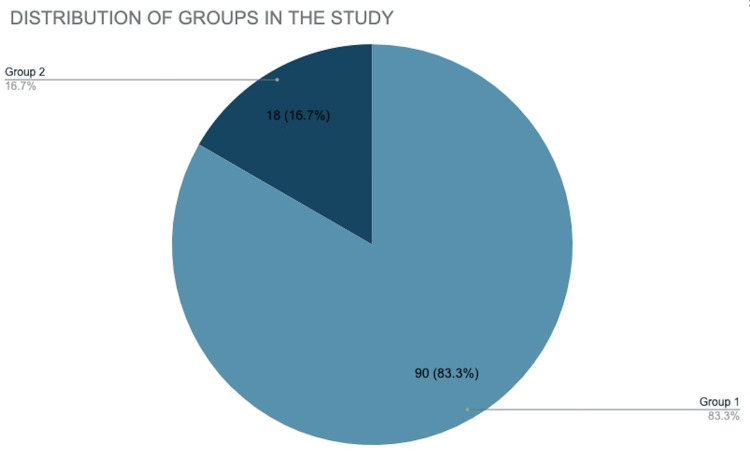
Distribution of patients among the two groups in the study Group 1-Lactational breast abscess (n=90) Group 2-Non-lactational breast abscess (n=18)

Of a total of 108 patients, 75% (n=81) belonged to the age group of 20-30 years and almost 14% (n=15) belonged to the 31-40 year age group (Table 1). Thus majority of the women were between 20-40 years, and only 11% (12) women were above the age of 41 years. Approximately 83.3% of the patients belonged to Group 1, and 16.7% of them belonged to Group 2 (Figure 1). The mean age of participants in the study was 26±2 years. There was no history of smoking or Type 2 diabetes mellitus or history of previous similar infections, thus eliminating the most common risk factors in Group 2. In contrast, a history of cracked nipples and nipple trauma was seen in 27 patients, accounting for a total of 33% of patients in Group 1.

**Table 1:** Age distribution of patients in the study (N=108)

Age Group( years)	Number of patients (N=108)
20-30	81
31-40	15
41-50	7
51-60	5

Table 2 shows a list of all the organisms along with their resistance patterns. *S. aureus* is the most common organism isolated (41.67% of total isolates), with both methicillin-sensitive and methicillin-resistant (MRSA) strains identified. MRSA demonstrated significant resistance to penicillins and cephalosporins and was susceptible to vancomycin and clindamycin, which remain effective treatment options. *Streptococcus pyogenes *(*S. pyogenes*) is the second most common pathogen, found in 13.89% of patients. *Escherichia coli* (*E. coli*) and *Enterococcus faecalis* (*E. faecalis*) are also significant pathogens, with the latter showing resistance to vancomycin. Anaerobic bacteria and mixed infections are present in 5.56% of patients, indicating that abscesses in some cases may involve polymicrobial infections. *Mycobacterium tuberculosis* was found in 1.85% of patients, indicating the need to consider tuberculosis mastitis in endemic areas. A total of 3.7% (n=4) of the total isolates were found to be coagulase-negative Staphylococci (CoNS), of which all four were resistant to methicillin, highlighting its persistent significance in breast infections. Fungal infections, primarily *Candida *species, were noted in a few non-lactating patients, highlighting the importance of considering fungal etiology in such populations. *Klebsiella pneumonia *(*K. pneumonia*) strains exhibited resistance to multiple antibiotics, including extended-spectrum beta-lactams, highlighting the growing concern of multidrug resistance in breast abscess infections. 

**Table 2:** Distribution of organisms and their resistance across the study ESBL: extended spectrum beta lactamase; MRSA: methicillin-resistant Staphylococcus aureus; MSSA: methicillin-sensitive Staphylococcus aureus

Organism	Total Isolates (n = 108)	% of Total Isolates	Aerobic/Anaerobic	Antibiotic Resistance
Staphylococcus aureus	45	41.67-MRSA (15%), MSSA (26%)	Aerobic	Penicillin resistance
Streptococcus pyogenes	15	13.89	Aerobic	Low resistance
*Escherichia coli* (ESBL-producing)	10	9.26	Aerobic	-
Enterococcus faecalis	8	7.41	Aerobic	Vancomycin resistant
Klebsiella pneumoniae	7	6.48	Aerobic	Beta lactams resistant
Pseudomonas aeruginosa	4	3.7	Aerobic	-
Staphylococcus epidermidis	4	3.7	Aerobic	Coagulase-negative, methicillin-resistant
Mixed anaerobic culture	6	5.56	Anaerobic	-
Mycobacterium tuberculosis	2	1.85	Aerobic	-
Fungal (including *Candida* species)	2	1.86	Mixed	-
No growth	5	4.63	-	-

**Figure 2:**
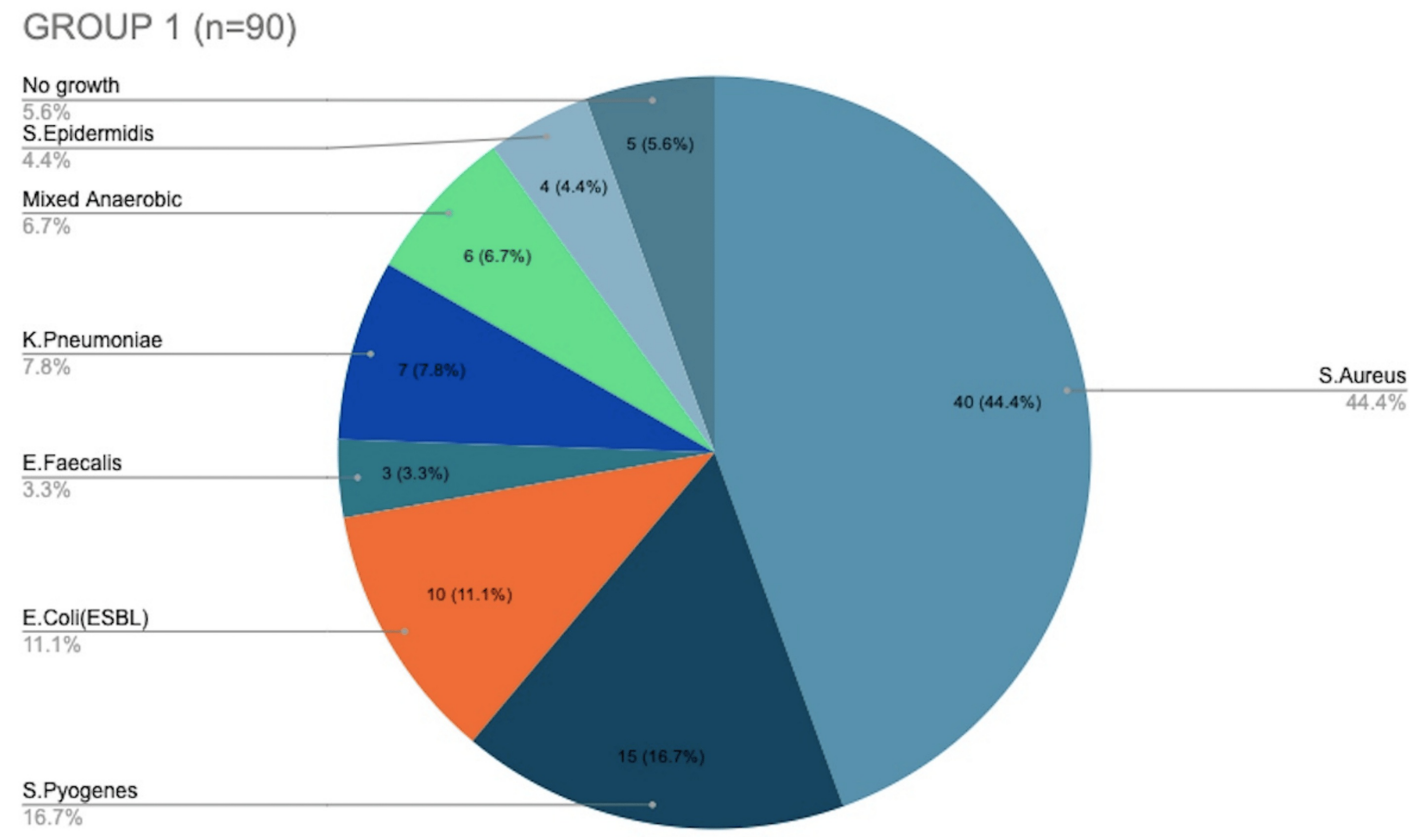
Distribution of microorganisms in Group 1

**Figure 3:**
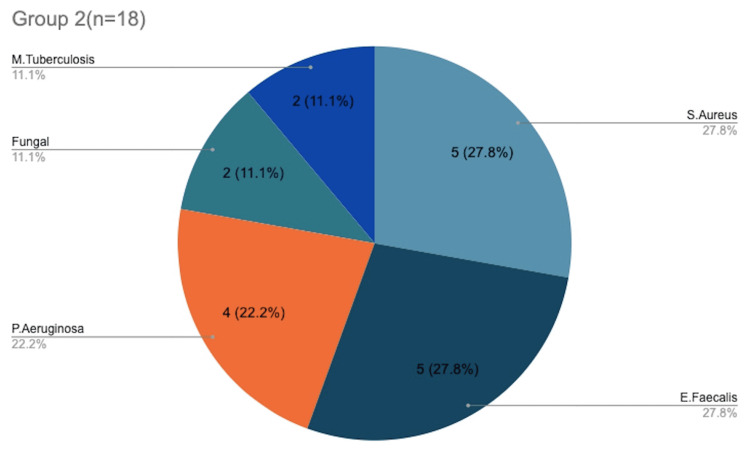
Distribution of microorganisms in Group 2

Figure 2 shows the distribution of microorganisms in Group 1, with* S. aureus* found in 44.4% of cultures and *S. pyogenes *infecting 16.7% of the patients. Of the total 108 participants, 10 were found to have *E. coli* infection and all of them were found in Group 1. Figure 3 shows the distribution of microorganisms in Group 2, with *S. aureus* and* E. faecalis* infecting five patients each (27.8%), and* P. aeruginosa *affecting four patients (22.2%) in total. A total of two patients were infected with *M. tuberculosis*, and both were found only in Group 2. After further confirmatory studies, both patients were started on anti-tuberculous therapy. Fungal elements were seen in two cultures and thus were taken up for further testing. *E. faecalis* was found in eight patients in both groups, of which two were resistant to vancomycin. A total of four isolates of *Staphylococcus epidermidis* were coagulase negative. They showed complete resistance to methicillin but showed sensitivity to second-line antibiotics like ciprofloxacin. On comparing the pathogen distribution between Group 1 and 2, although MRSA was the most common pathogen overall, Group 1 saw more *K. pneumoniae* and ESB, whereas Group 2 saw more *E. faecalis* followed by* P. aeruginosa*.

## Discussion

Breast abscesses are most commonly associated with mastitis, a bacterial infection of the breast tissue that typically occurs during breastfeeding. Mastitis can occur when bacteria, such as *S. aureu*s, enter the breast through cracked or damaged skin on the nipple. This is especially common in the early stages of breastfeeding, when issues like nipple trauma, milk stasis, or inadequate emptying of the breast are more frequent [1]. It can also occur in patients with increased stress, poor sleep patterns, poor immunity or if the infection remains untreated for long periods of time [3, 6-9]. On the other hand, a history of smoking, higher BMI, African-American race and Type 2 diabetes are all risk factors for non-lactational breast abscess [10, 11]. Presents as a localized swelling, redness, pain, and a palpable lump that may fluctuate in consistency, sometimes accompanied by fever. Generally managed with antibiotic therapy, ultrasound-guided incision and drainage is required in advanced cases.

The findings of this retrospective study align with the results of other similar studies conducted worldwide. A study by Ramakrishnan et al. in India found that *S. aureus* was the most common pathogen isolated from breast abscesses, with a similar prevalence of MRSA [12]. In a study by Lodhi et al. in Pakistan, *S. aureus* was also the predominant pathogen, although *Streptococcus *species and gram-negative organisms were less commonly isolated compared to our cohort [13]. The lower frequency of *Streptococcus* species in the Pakistan study may be attributed to the differing healthcare settings and geographic location, which may influence the microbial flora. The high prevalence of MRSA in the current study is consistent with the increasing global trend of antimicrobial resistance. MRSA is a significant cause of healthcare-associated and community-acquired infections, including breast abscesses. Although MRSA is susceptible to vancomycin and clindamycin, the emergence of resistance to Clindamycin has been documented in other studies [14]. In this study, we found that the majority of MRSA isolates remained susceptible to these antibiotics, which is encouraging, but vigilance in monitoring resistance patterns remains essential.

Bartolomé et al. conducted a study where anaerobic bacteria were the most frequent pathogen isolated in non-lactating women, followed by *Staphylococcus* species, mostly affecting the lactating women [15]. They suggested that smoking was a major risk factor, as a significant proportion of non-lactating women were smokers in their study. However, contrary to this, a study by Rizzo et al. found no direct correlation between breast abscess and smoking history [11]. All the patients in our study had no history of smoking, type 2 diabetes mellitus or history of similar infections. Much like similar studies, we also found* Streptococcus*,* E. coli*, and *K.* *pneumoniae* in our study [1, 12].* S. pneumoniae* and *S. pyogenes* are known to cause infections in soft tissues, including abscesses, and can occasionally present in breast abscesses, particularly in smokers and those with a previous history of breast infection. A study conducted in Spain in 2020 reported Streptococcus in about 35% of the patients, much higher than our cohort of 14%, thus elucidating the importance of regional variation [16].

CoNS, traditionally considered benign skin flora, has emerged as a significant pathogen in breast abscesses, emphasizing its role beyond mere contaminants. Furthermore, it was observed that CoNS accounted for 16% of positive cultures as compared to 3.7% in our study, underscoring their persistent relevance [17]. Although in our study the most common CoNS species was* S. epidermidis*, another study showed that most of its isolates were *S. lugdunensis* [18]. These findings collectively suggest that CoNS should be regarded as potential pathogens in breast abscesses, necessitating appropriate clinical attention and management. Approximately six isolates were found to have mixed anaerobic infections seen in lactating women. These infections complicate treatment, as they require broader-spectrum antibiotics sometimes for longer durations. They can also lead to higher recurrence rates, warranting tailored antimicrobial therapy post-culture to address the mixed pathogen profile effectively.

We found a relatively higher incidence of *E. coli* (9.26%) in our cohort as compared to the other studies, which reported a higher incidence of *Proteus* and *Acinetobacter* [1, 12]. Fungal infections, particularly caused by *Candida* species, were also observed in a small percentage of cases in our study. Although *Candida* is known to contribute to mastitis and sore nipples in lactating women, in our study, these were found only in non-lactating women, which may be due to presence of skin flora or a history of vaginal yeast infection. While fungal infections in breast abscesses are rare, they should be considered in patients with predisposing risk factors, as they may require antifungal therapy in addition to antibiotics. Our cohort showed similar results for *S. aureus* when compared with existing studies, although there was a significantly higher incidence of *E. coli* and *Enterococcus* in our study, drawing attention to the recent change in trend for causative agents for breast abscess. Our study's limitations include a relatively small sample size, limited information on clinical outcomes and follow-up, the absence of molecular typing for MRSA and ESBL, and its retrospective design, which may introduce selection bias.

## Conclusions

*S. aureus* was the most predominant pathogen in our study, followed by *S. pyogenes* and* E. coli*. The growing incidence of MRSA underscores the importance of appropriate antibiotic selection based on microbiological data. Regional variations in the microbiological profile of breast abscesses suggest that local healthcare practices and patient demographics play an important role in determining the causative organisms. Further studies are needed to monitor the changing landscape of microbial etiologies and resistance patterns, particularly in light of emerging multidrug-resistant pathogens.
